# Design and Engineering of Miniproteins

**DOI:** 10.1021/acsbiomedchemau.2c00008

**Published:** 2022-04-28

**Authors:** Katarzyna Ożga, Łukasz Berlicki

**Affiliations:** Department of Bioorganic Chemistry, Wrocław University of Science and Technology, Wyb. Wyspiańskiego 27, 50-370 Wrocław, Poland

**Keywords:** miniproteins, peptides, tertiary structure, folding, noncanonical amino acids, computer-aided design

## Abstract

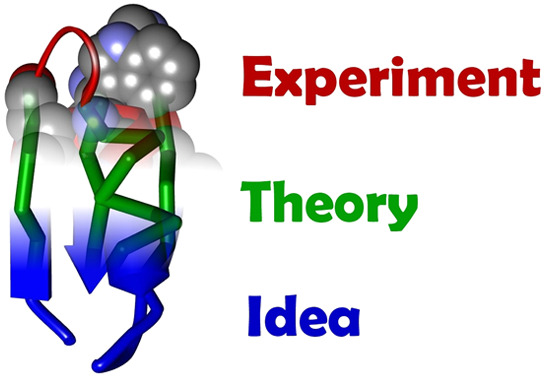

The potential of miniproteins in the biological and chemical sciences is constantly increasing. Significant progress in the design methodologies has been achieved over the last 30 years. Early approaches based on propensities of individual amino acid residues to form individual secondary structures were subsequently improved by structural analyses using NMR spectroscopy and crystallography. Consequently, computational algorithms were developed, which are now highly successful in designing structures with accuracy often close to atomic range. Further perspectives include construction of miniproteins incorporating non-native secondary structures derived from sequences with units other than α-amino acids. Noteworthy, miniproteins with extended structures, which are now feasibly accessible, are excellent scaffolds for construction of functional molecules.

The increasing complexity of the structures and functions of molecules is one of the major challenges in chemistry and related sciences. In nature, proteins play a pivotal role due to their wide repertoire of functions and great diversity of three-dimensional structures. Therefore, the rational design and engineering of proteins have gained a lot of attention. In particular, miniproteins, i.e., proteins with molecular mass not exceeding 10 kDa exhibiting a well-defined three-dimensional structure, have recently become an emerging class of molecules.^1−5^ They combine several features that make them valuable and attractive compounds. First, modern computer-aided techniques allow efficient design of new structures.^6−9^ Second, miniproteins can be obtained using a solid-phase peptide synthesis approach or overexpressed in cells. The first option provides the opportunity to introduce any chemical modifications and thus robust engineering of any fragment of a molecule. On the other hand, the second option gives access to a high number of various mutants and methodologies, allowing selection of miniproteins with the highest biological activity (e.g., phage display techniques). Third, miniproteins can be readily analyzed structurally by a number of techniques developed in peptide/protein sciences including circular dichroism (CD), nuclear magnetic resonance (NMR), fluorescence spectroscopy, and X-ray crystallography. Fourth, the obtained structures can be significantly diversified considering both overall three-dimensional arrangement and chemical properties of individual functional groups that could be placed at any chosen part of the molecule. Significant effort has already been made to obtain structures incorporating noncanonical amino acids, both by rational design^10^ and combinatorial libraries.^11^ Therefore, great possibilities of engineering the properties of miniproteins are available. Fifth, this robust class of molecules can constitute a source of scaffolds for the construction of functional compounds. Numerous applications, in particular, in medicinal chemistry, have already shown the potential of miniproteins.^12,13^ Engineered miniproteins have been shown to exhibit anticancer,^14−17^ antibacterial,^18,19^ and antivirus activities.^20,21^ The two recent examples have shown picomolar inhibitors of SARS-CoV-2 S-protein/human ACE2 protein interaction^22^ and potent PD-1 agonists constructed using miniprotein scaffolds.^23^ Miniproteins were also utilized to install various types of catalytic activity. Examples include short β-hairpin with hydrolase activity,^24^ heme-cage-forming β-sheets with peroxidase activity,^25^ and recently reported covalently bound helical bundle catalyzing acyl transfer reaction.^26^

In this Perspective article, we will focus on the development of new miniproteins. Similarly to native proteins, the structures of miniprotein can be stabilized by any combination of three major types of interactions, namely, hydrophobic core, covalent links, e.g., disulfide bridges or thioether links (covalent core),^27^ and metal complexation. Here, however, we will place emphasis on monomeric miniproteins stabilized by a hydrophobic core and containing an aliphatic backbone due to the robustness of the design and analysis approaches for this class of miniproteins as well as their straightforward synthesis (i.e., up to 50 amino acids and with no additional modification steps being necessary). Initially, miniprotein construction methodologies were based on the preferences of individual amino acid residues for a particular secondary structure. These approaches were able to provide several miniproteins, however, usually not fully defined or appropriately characterized. The other approaches make use of engineered fragments of native proteins. As time passes, the accuracy and possibilities of computer-aided methodologies have been increasing, and the de novo design of miniproteins with atomic precision has recently become available. Therefore, after 30 years, the research finally reached a level that allows for efficient construction of any desired miniprotein molecule.

There are three ways of the tertiary fold design that are dominating from the very pioneering reports up to the most recent ones. They range from bottom-up to top-down approaches: (a) de novo design using the principles of folding (assembly of secondary structures); (b) experimental data-based de novo design (from topology to sequence); (c) alteration of the existing sequences of known miniproteins (engineering).

## Early Knowledge-Based Design

The first approach to the tertiary structure forming peptides was based on conformational probability parameters for a single amino acid residue. The first foundations described in the works of Chou and Fasman^28^ and Levitt^29^ based on the set of X-ray protein structures, which allowed quantitative evaluation of the preferences of amino acid residues to form or break the particular secondary structure. For example, Glu, Ala, and Leu residues are strong α-helix formers; β-sheets are predominantly induced by Met, Val, and Ile residues, whereas Pro and Gly normally terminate (break) both of these secondary structures.^28^ The nucleation (initiation) of the secondary structure is facilitated by the presence of a cluster of four or five strong forming residues. Furthermore, a different distribution of the amino acid residues at the N- and C-termini (boundaries) of the secondary structures was discovered. Proper placement of charged residues along the helix can greatly stabilize its fold.^30^ Negatively charged residues should be incorporated into the N-terminal part and positively charged residues near the C-terminus, which allows proper electrostatic interaction with the α-helix dipole (positive pole at the N-terminus and negative pole at the C-terminus). The proposal of the three-dimensional structure is the initial step in the miniprotein design based on these rules. Subsequently, the sequence is proposed using parametrized prediction methods. The construction of nucleic-acid-binding 34 residue polypeptide folding into the ββα tertiary structure is one of the first examples (Table 1, sequence **1**).^31^ The very low solubility suppressed the elucidation of the high-resolution structure in solution; however, the CD spectrum indicated the presence of a considerable amount of organized structure. Another example of this approach is the β-bend (short antiparallel sheet) formed from the repeating sequence of amino acid residues that promote the strand and that break (Pro-Gly) the strand.^32^ In this case, folding to an extended structure was also confirmed only by CD.

**Table 1:**
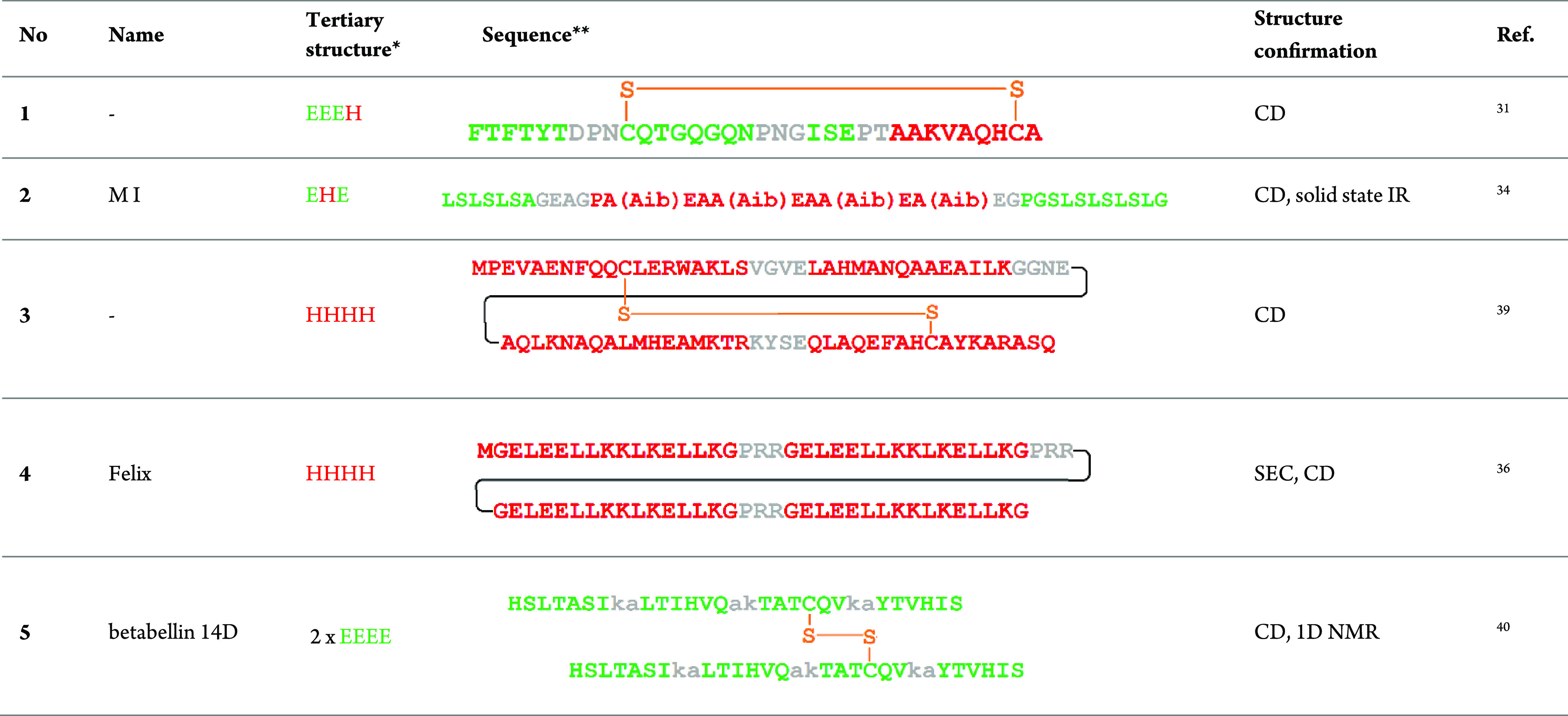
Selected De Novo Designed Sequences without High-Resolution Structure,,,,

* Simplified representation of the tertiary structure, where E denotes the extended conformation and H denotes the helical conformation.

** Residues marked in green, red, and gray denote strands, helix, and loops/turns forming residues, respectively. Orange lines represent disulfide bonds. Small letters denote analogous d-amino acids. The long sequences (**3** and **4**) were split into two parts connected by a black line.

**Figure 1:**
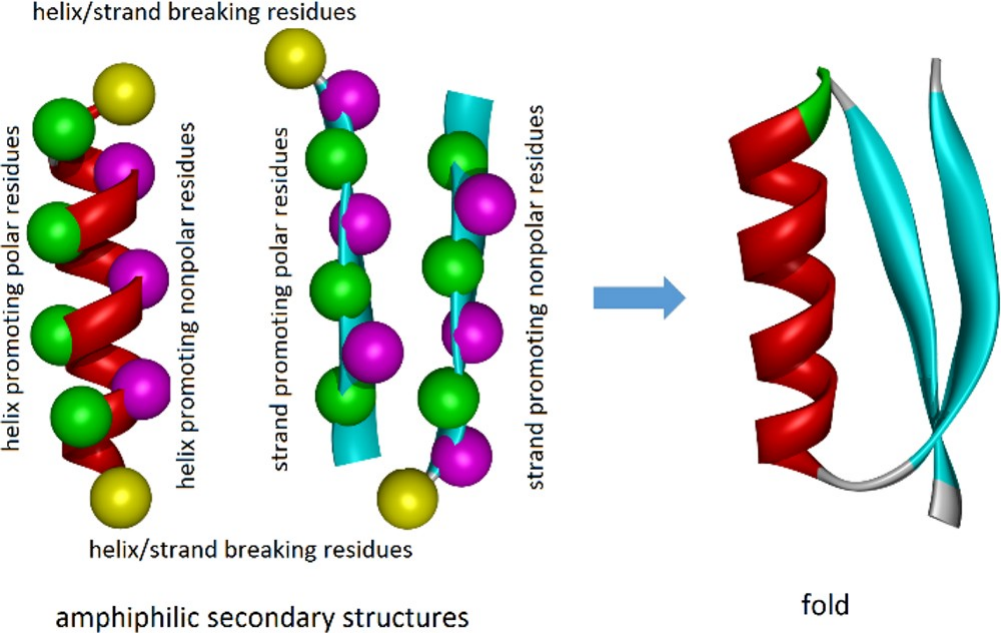
Simplified representation of the design process presented in the early studies. The tertiary fold is the result of the linkage of the amphiphilic secondary structures. The sequences of the secondary structures were designed according to the conformational preferences of the amino acids. The peptide backbone is presented in a ribbon diagram, and side chains are presented as balls.

The alternative concept of the de novo design of the tertiary structure was based on the understanding of the protein folding process at that time, which was oversimplified to two steps: (a) formation of the secondary structure and (b) folding into the tertiary structure. As the driving force for folding into a stable tertiary structure is the formation of a hydrophobic core with the release of the solvation entropy, and the construction of folding units should be possible by the assembly of amphiphilic segments with a secondary structure-forming potential (Figure 1).^33^

According to this assumption, several α/β structures were designed. An example is the βαβ fold (Table 1, sequence **2**).^34^ The β-structure-forming fragments were created by alternating hydrophilic and hydrophobic amino acid residues (Ser and Leu) in the sequence, while the stable amphiphilic helix was achieved by incorporation of the 2-aminoisobutyric acid (Aib) residue into the hydrophobic core together with the Glu and Ala residues. The lengths of the secondary structure were chosen on the basis of the typical lengths of these segments in native βαβ-fold-containing proteins. The CD spectrum indicated a highly ordered structure in solution, which was further confirmed by infrared spectroscopy in the solid state.

A similar approach was chosen for the construction of covalently bound 4-helix bundles (Table 1, sequence **3**).^35,36^ In one of the studies, initially, the sequence of an amphiphilic single helix was designed based on helix bundles found in native proteins, such as cytochrome *c*′, with alternating Leu and charged Glu/Lys residues only.^35^ Additionally, the stabilization of the helix dipole was included as described above.^30^ The sequence of the full-length protein forming the bundle was then designed by incorporating four repeats of the helix sequence spaced by the Gly-Pro-Arg-Arg linker. The monomeric, globular, and compact structure of the obtained miniprotein was confirmed by size exclusion chromatography (SEC) and CD. The GuHCl denaturation monitored by the CD signal confirmed cooperative folding. Moreover, the conformational stability was much higher for the bundle than for the single helix or double helix. The helicity of the helical fragments was also confirmed by NMR, but the overall structure could not be resolved due to the symmetry incorporated into the design (only 16 amides were present, which is the length of one helix and the turn segment).^37^

The other pioneering study aimed to differentiate the helix bundle sequence for two reasons: (a) to facilitate the NMR analysis and structure calculation and (b) to design a more native-like sequence.^36^ Although the “Felix” protein had 79 amino acid residues, it can still be considered a miniprotein due to the design concept (Table 1, sequence **4**). The sequence was designed to fold into an antiparallel four-helix bundle and not to be homologous to any known protein sequence. In addition, the disulfide bridge connecting the first and the last helix was introduced to stabilize the overall fold. The introduction of a single Trp residue designed to be buried in a hydrophobic core of the structure was another new property. Helical fragments were designed to be amphiphilic as in above-described designs with the use of charged residues, uncharged hydrophilic residues, large hydrophobic residues, and Ala residues in a 1:1:1:1 ratio. Initially, the detailed sequence of helices and turns was assessed with the use of Chou and Fasman prediction programs (which classifies residues just in the three groups: as strongly forming, forming, or breaking the secondary structure).^28^ Later, the more sophisticated sequence prediction method was applied, which was based on the conformational preferences of residues to the specific position in the secondary structure relative to its N- and C-termini (based on the abundance of residues in a particular position in the native secondary structures).^38^

The N-terminal positions of the helices in Felix were Gly, Asn, and Ser residues (the most favored in native helices), and the C-terminal positions were Ser, Gly, Arg, and Gln. Gly residues are the most favored C-cap in natural proteins, and Thr, Asn, Tyr, and Cys residues were introduced most often in the middle. As in previous studies, hydrophobic residues were used to form the interior of the designed structure. On the surface of the structure, 23 charged residues were introduced, so that they can form salt bridges, and they were distributed to stabilize the helix dipole. Between the helices, one- or two-residue turns taken from the native structures of myohemerythrin and hemerythrin were incorporated. Before the synthesis, the physical space-filling model was built, and the sequence was optimized to remove overlaps and fill holes. For the first time, the concept of negative design, i.e., excluding of not-desired folds, was also proposed and applied. The alternation of hydrophilic and hydrophobic residues proven to promote β-structures was avoided in the sequence.^34^ To avoid the formation of a hairpin or a very long helix oligomerizing in the coiled-coil structure, (a) the very strong helix breaker was used in turns and (b) the hydrophobic stripes of the individual long helices were not positioned to produce the hydrophobic core of the coiled coil. The Felix structure was confirmed by SEC and CD as monomeric with 50–65% of α-helical conformation, as observed for native four-helix bundles. The other tertiary folds addressed by the negative design could be ruled out by the experimental data.

The work on conformational preferences of amino acids within the secondary structure was also used to build the first β-sheet structure—betabellin.^40^ The β-sheet is less modular than the α-helix, requires more cooperativity to fold properly, and has a greater tendency to form aggregates in solution. The betabellin structure was designed to consist of two amphiphilic β-sheets held together (dimerized) by interaction of their hydrophobic surfaces. The sequence was characterized by the palindromic pattern of polar (p), nonpolar (n), end (e), and turn (t,r) residues: epnpnpn-tt-npnpnp-rr-pnpnpn-tt-npnpnpe. The β-structure fragments were designed according to the conformational preferences rule (with alternating polar and nonpolar residues). The turns tt and rr comprise d-Lys-d-Ala and d-Ala-d-Lys fragments, respectively, which were calculated through molecular dynamic simulations as favoring folding into type I′ turn.^41^ The first efforts produced 32 residue long sequences of betabellin 12S, which was proven by CD and NMR to form a noncovalent dimer, but the second version, betabellin 12D, where the two β-sheets are connected by a disulfide bridge was aggregating in water and probably was present in two distinct conformation in solution. In the next study, betabellin 14 was designed by mutation of polar residues to switch from −2 to +10 net charge. Unlike betabellin 12S, betabellin 14S exhibited the typical CD spectrum of random coiled proteins. However, betabellin 14D (the analogue with the disulfide bridge, Table 1, sequence **5**) showed good solubility and the characteristic of the CD spectrum common for β-sheet with a maximum around 195 nm and a minimum around 220 nm and cooperative unfolding during thermal denaturation. These observations were also confirmed by 1D NMR, which was much more dispersed for 14D. These findings proved that a binary pattern of polar and nonpolar residues may be sufficient to induce the folding of an α-helical structure^42^ but not necessarily a β-sheet.

## Structure-Based Design

In all of the reports presented so far, one major obstacle was encountered; i.e., the tertiary structures were not supported by the high-resolution structural data, e.g., calculated from NMR-derived constraints or X-ray diffraction. As most of them provide strong experimental data that support most of the expected structural features, obtaining three-dimensional structures could reveal some important differences from the design. Such data would also support an iterative cycle of design, structure determination, and redesign to obtain desired and stable miniproteins.

**Figure 2:**
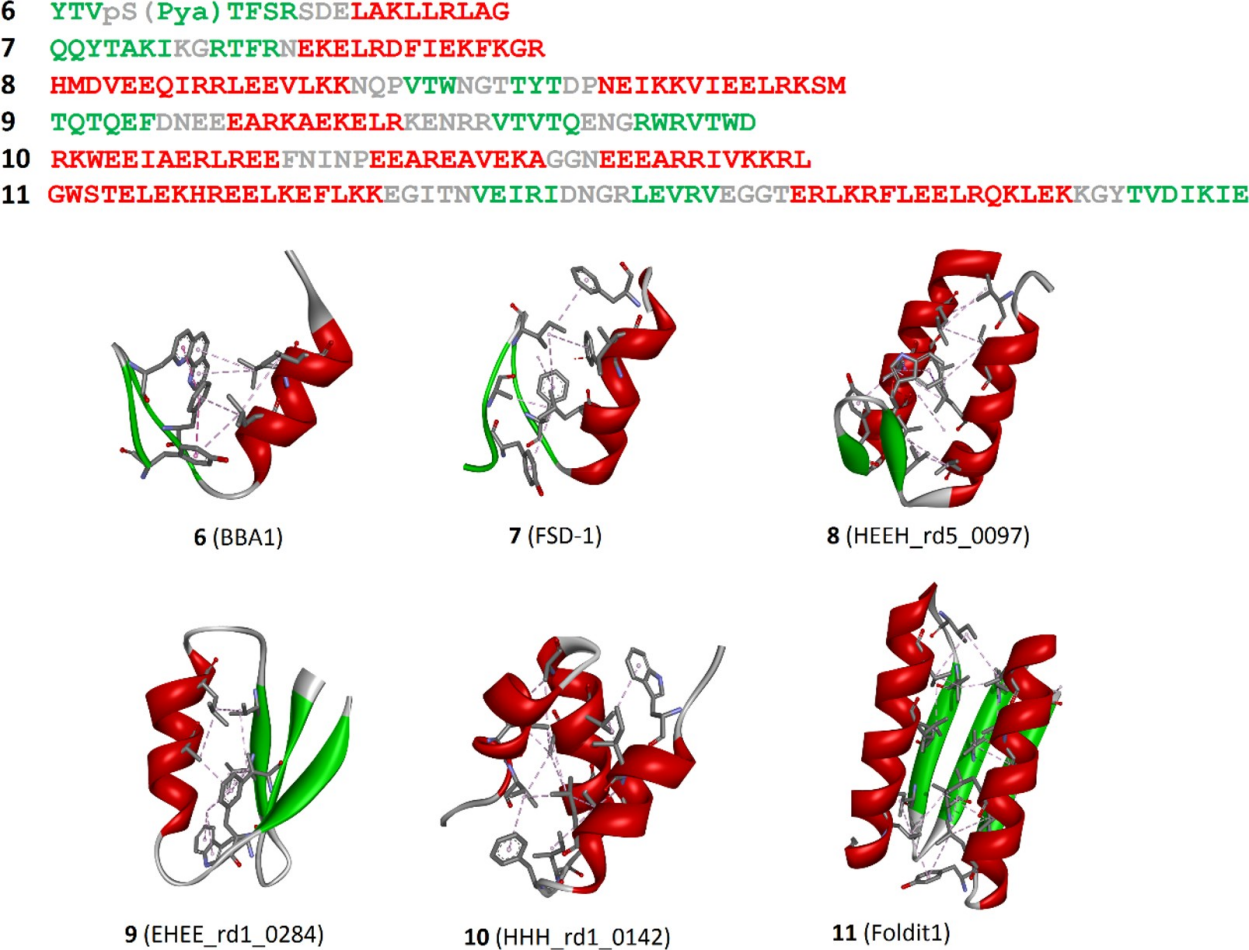
Sequences and NMR-derived structures of chosen de novo designed miniproteins. The peptide backbone is presented as a ribbon. The red color denotes the α-helix, green color denotes the extended structure, and the gray color denotes the loops and is consistent with the coloring of the sequences. The side chains that form the hydrophobic core are presented as sticks, and the interactions between them are depicted by dashed pink lines. All structures are present in the PDB database under the following codes: **6**, 1hcw; **7**, 1fsd; **8**, 5uyo; **9**, 5up5; **10**, 5uoi; **11**, 6mrr.

The helix–loop–helix-forming peptide ALIN stabilized by a single disulfide bridge was the first miniprotein with NMR-resolved structure.^43^ The design strategy was very simple: choose sequences known to produce stable amphiphilic helices and connect them with a sufficiently flexible loop with the GTSG sequence in the center and without proline residues to avoid *cis*–*trans* isomerization. The hydrophobic interface between the helices was created from four hydrophobic residues (Val and Leu). Additionally, the interaction within hydrophobic faces of the helices was stabilized by introducing a disulfide bond. The CD analysis confirmed the helical structure, and cooperative folding of ALIN and SEC indicated its monomeric state. In contrast to previously reported miniproteins, the NMR spectrum showed sharp signals, which allowed peak assignment and structure calculation on the basis of NOE-derived restraints. ALIN adopts the helix–loop–helix conformation in solution as designed with a very well-determined helical region and more fluctuating termini and loop regions. The spatial arrangement of the helices is also well-defined. In addition, the H/D exchange rates of the amide protons indicated the presence of stable hydrogen bonding within the helices. The next study aimed to obtain a zinc-finger-like ββα structure with the following assumptions addressing limitations of previous reports: (a) absence of any cross-links such as disulfide bridges or metal binding; (b) monomeric structure; (c) water solubility; (d) short sequence.^44^ The native zinc finger structure depends on the presence of metal cations; however, large hydrophobic residues clustered in the core and conserved among all structures (typically Tyr, Phe, and Leu) could be optimized to obtain a metal-independent tertiary fold. Another justification for the choice of a topology was that the CD spectra of the zinc fingers were well-known and characteristic, so the secondary structure content of the designed sequences could be easily assessed. The design process included four rounds and starts from the native sequence of the zinc finger, and because of that, it can also be considered as one of the first trails to obtain miniproteins by protein engineering (described below). It is also the first study in which the iterative rational design was presented. In the first generation, the turn between β-strands was redesigned to form a type II turn (by mutation to Pro-d-Ser),^45^ and the loop connecting the β-hairpin with an α-helix was shortened by 4 amino acid residues. Furthermore, the metal coordination site was changed from Cys_2_His_2_ to His_2_Pya (Pya: 3-(1,10-phenanthrol-2-yl)-l-alanine) to include the reporter group. The stabilization of the secondary structures on the basis of conformational preferences of amino acid residues was also included in the second generation of sequences. The Ala residues present in the β-hairpin were mutated into Thr residues, whereas the Thr and Ile residues in the helix were changed to Ala and Leu. The first two generations adopted a tertiary fold, but it was dependent on the presence of metal. In the third generation, the type II turn (Pro-d-Ser) was mutated into a sequence that promotes the type II′ turn (d-Pro-Ser)^45^ which, based on native protein structures, can promote more the β-hairpin formation. This change resulted in a metal-independent tertiary fold as studied by CD at different metal concentrations. However, NMR analysis indicated the presence of two distinct conformations in the 1:1 ratio as a result of the *cis*–*trans*d-Pro isomerization. In the next generation, the amount of *cis* isomer was reduced to 25% by mutation of the residue preceding d-Pro from His to Val. These four rounds of design succeeded in producing a 23 residue BBA1 peptide (Figure 2, sequence **6**) that is very soluble and monomeric in solution, allowing detailed NMR analysis and structure elucidation. The BBA1 structure proved to be very similar to natural zinc fingers, however, more open. The stability of the structure results from the formation of a hydrophobic core: most long-range NOE contacts came from residues Tyr1, Phe8, and Leu14. BBA1 was then redesigned not to contain a synthetic Pya residue in the hydrophobic core.^2^ The mutation of Pya into Tyr or Trp residues caused increased flexibility in the sheet region of the structure. This problem was eliminated by adding charged residues on the exterior of the β-hairpin to destabilize the unwanted fold, which is the next example of negative design. The d-Pro residues were proven as nucleating the β-hairpin formation in another study, where the d-Pro-l-aa sequence promoting the type II turn was used in the construction of a β-sheet structure of Beta-4.^46^ Unlike betabellin, Beta-4 was 26 amino acids long, soluble, and did not aggregate, allowing the detailed structure to be solved using NOE-derived restraints.

## Computational De Novo Design

**Figure 3:**
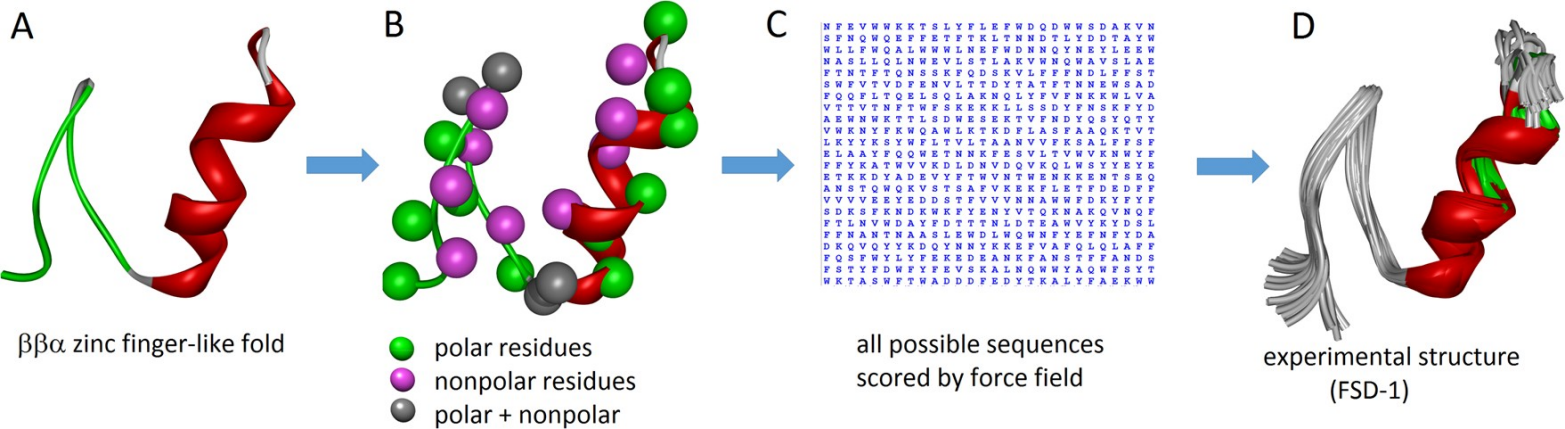
Scheme of the computational design of FSD-1. Chosen backbone conformation presented in ribbon diagram (A). The possible sequences were limited by classifying residue positions shown as balls as the core, surface, or boundary (B). All of the left sequences were computationally scored (C), and the structure of the lowest energy one was experimentally confirmed (PDB 1fsd) (D). The side chains are shown as sticks.

Modern de novo design became possible with the development of computational resources and methods. The first foundation for the new era of computational design was established quite early in 1997 by Dahiyat and Mayo,^47^ who introduced the fully automated protein design to obtain a zinc-finger-like ββα fold. The reported methodology begins with the choice of the backbone fold (Figure 3A) and then the choice of a sequence that could stabilize the target structure. The selection is based on the calculated potential of interactions between side chains and side chains and backbone (force field).^48^ The number of sequences possible for the designed 28 residue backbone was reduced by a classification of the amino acid position to the core (Ala, Val, Leu, Ile, Phe, Tyr, and Trp), the surface (Ala, Ser, Thr, His, Asp, Asn, Glu, Gln, Lys, and Arg), and boundaries (combined set from core and surface) (Figure 3B). The number of sequences was still 15 orders of magnitude higher than the number accessible for testing experimentally (e.g., with combinatorial libraries). However, all of them, together with a discrete set of side chain rotamers, were computationally screened and scored using force field potentials (Figure 3C). The CPU time was limited to only 90 h with the use of properly designed algorithms (such as DEE for rotamer elimination^49^). The optimal sequence called FSD-1 was experimentally validated (Figure 2, sequence **7**, Figure 3D). CD analysis revealed a spectrum typical for zinc finger proteins and the cooperativity and reversibility of folding with a melting point at 39 °C. The detailed structure was calculated based on NOE-derived restraints, and the structure of the backbone was almost identical to the structure of the native zinc finger protein Zif268^50^ but showed a well-defined hydrophobic core built from seven large hydrophobic residues.

In the more recent works of Baker and co-workers, the concept of negative design was brought up again.^51^ The main statement of the design process was that the miniprotein will properly fold if the energy landscape of folding is funnel-shaped; i.e., for the particular sequence, there is an energy gap between the desired structure and any other structures (which can be calculated by independent Monte Carlo simulations from the extended structure). To destabilize undesired folds, the focus was more on local interactions independent of the amino acid sequence but rising from the chirality of the polypeptide chain, as nonlocal interactions vary significantly with even small changes in the tertiary structure.

**Figure 4:**
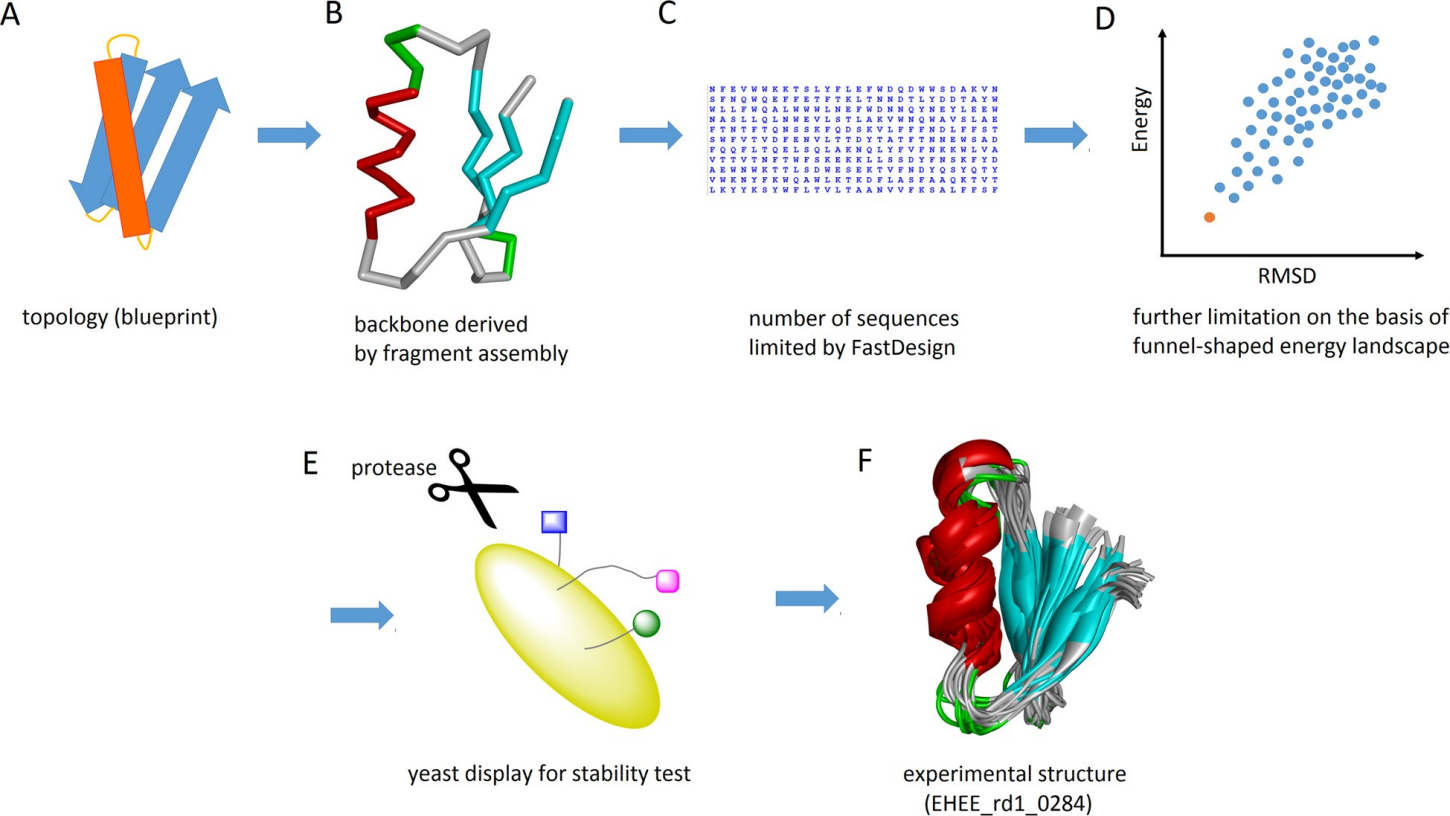
Scheme of the computational design developed by Baker and co-workers. The design starts with a simplified tertiary topology (A). The orange tube represents and α-helix, and the blue arrows are β-strands. The real backbone conformation presented as C_α_ stick is derived by fragment assembly (B). The red color denotes α-helix, blue denotes β-strands, green are turns, and gray are coils. Possible sequences were obtained using the FastDesign protocol (C) and evaluated by calculating the folding energy landscape (D). The most stable designs were identified experimentally by protease digestion of yeast displayed libraries (E) and their structure resolved by NMR (PDB: 5up5) (F) as presented as a ribbon with colors analogous to (B).

In their first study, the set of rules relating secondary structure patterns to protein tertiary motifs was established by combined analysis of native protein structures in the PDB database and de novo folded structures using Rosetta software. The calculation involved several small tertiary α/β motifs within which the length of the strands, helices, and loops varied. These calculations revealed that the mutual orientation of the secondary units is very strongly dependent on their lengths. Analysis revealed the three fundamental rules independent of side chains to describe the junctions between secondary structures: ββ rule, βα rule, and αβ rule. The ββ rule indicates that the relative positioning of the strands in a ββ motif depends on the length of the loop. In the βα motif, the helix position is determined by both the loop length and the direction of the side chain of the last residue in the strand. In the αβ motif, the side chain of the first residue of the strand should point away from the helix. According to these rules, the secondary structure length (structure patterns) was derived for five different protein topologies. The desired structures were then stabilized by favorable nonlocal interactions (between amino acid side chains) using the RosettaDesign protocol^52^ (which chose the best amino acid sequence for the designed fold) (Figure 4C) and filtered according to relative energy and packing. For the filtered sequences, hundreds of thousands of independent Rosetta folding simulations were performed to calculate the energy landscape. Approximately 10% of the designed sequences had, in fact, a funnel-shaped energy landscape (Figure 4D) and were expressed in the bacterial system for experimental validation. The SEC indicated the monomeric miniproteins for each of the five desired folds. All of them were very soluble and had a CD spectrum typical for α/β proteins and a high melting point. The NMR-derived structures (Figure 4F) were highly consistent with the computational design models. The described approach was also used successfully in the consecutive study to obtain ferredoxin-like and Rossmann 2 × 2 proteins.^53^

Later, together with the development of Rosetta software protocols, the backbone structure was produced by a slightly different approach but still consistent with a funnel-shaped folding energy landscape. The approach called fragment assembly is a Monte Carlo simulation during which short backbone fragments are selected from a database of crystal structures to match the desired topology (called a blueprint) (Figure 4A,B). The other stages of the design process remain similar. The usefulness of the described approach to produce new custom-made miniprotein structures was proven in several studies (Figure 2, sequences **8**–**10**),^22,54,55^ and possible applications in medicinal chemistry were also reported.^22,23^ However, it should be underlined that the success of this methodology depends on high-throughput screening methods for experimental validation. In most cases, the number of sequences studied in detail was limited by preliminary stability studies. Designs were displayed on the surface of the yeast and treated with proteases (Figure 4E). The most resistant structures were then overexpressed in *Escherichia coli* and structurally evaluated. On the contrary, most of the methods for the design of miniproteins described above required only a few sequences to be tested.

The method based on Rosetta software protocols starts with the design of topology (tertiary structure) and is one of the best described and most successful methods in obtaining new folded proteins. There are, however, other strategies for computational design without the target structure in mind.

The fascinating example is provided by the Foldit game.^56^ This another idea from Baker and co-workers was to decrease the computational cost of de novo folding by utilization of non-expertise citizens and their intuition. Foldit is a free online game that was initially designed to support protein structure prediction problems; however, it was recently also successful in designing new stably folded proteins. The game starts with a fully extended polyisoleucine backbone (60–100 residues), and the users have to fold it into a compact tertiary structure together and change the sequence to minimize the Rosetta scoring function and to satisfy several rules; e.g., some proportion of residues has to be buried inside the structure, or glycine and alanine residues cannot form secondary structures. In contrary to automatic Rosetta sampling protocols, the biggest advantage of the game is that the design path of Foldit users tends to escape from local energy minima and explore new regions (during the design the structures often undergo large increase in energy). Some of the user-designed structures were solved experimentally and proven to be monomeric, compact in solution, and highly similar to the models. The examples include Foldit1 (Figure 2, sequence **11**), Peak6 (PDB: 6mrs), or Ferredog-Diesel (PDB: 6nuk).

The other methodology is inspired by evolution strategies. The SEWING (structure extension with native-substructure graphs) protocol combines pieces of naturally existing proteins in a process similar to recombination of genes.^57^ The idea behind is simple: the C-terminus of one fragment from the library fits to the N-terminus of another fragment. The combination of substructures should not produce steric clashes and should conserve loner-range tertiary interactions. The models obtained are then minimized using Rosetta scripts. Several of the miniproteins designed by SEWING were experimentally proven to be well-folded and highly stable with melting point temperatures above 50 °C. Two of them, CA01 and DA05, were hyperstable with the melting point temperature observed only in the presence of 5 M GdHCl, and their structures were solved in high resolution (PDB: 5e6g and 2n81, respectively). DA05 was further utilized as a scaffold to introduce enzymatic activity.^58^ Unfortunately, the SEWING protocol was used to date only to design helical proteins.

## Protein Engineering

Protein engineering was developed simultaneously to the first reports on de novo miniproteins and, contrary to de novo design, is based on changing given primary sequence of proteins by replacing amino acid residues while retaining the folding propensity.^59^

Protein engineering was based on the observation that many natural proteins are quite tolerant to mutations, and many different sequences can also be compatible with the same fold.^60^ The example of binary patterning of polar and nonpolar amino acids, where the massive sequence alteration still retained the same folding propensities, shows this dependence. The study of the four-helix bundle (73 residues) where the position of the hydrophobic and hydrophilic residues in the sequence was explicitly specified, but the specific identities of the side chains were randomly varied, confirming that, in most cases, such alterations do not have a significant impact on the overall fold.^42^ However, this may not apply to smaller miniproteins or β-structures, as observed in the modified version of de novo designed betabellin.^40^ One of the earliest examples of miniproteins engineered from native sequences were BBA peptides constructed by Imperiali and co-workers and described above.^44,61^ There are also examples of very short microproteins such as the peptide that contains WSXWS motif and stabilized by π-cation interaction^62^ or very short peptides (up to 10 residues) folding into short hairpins.^63−65^

**Figure 5:**
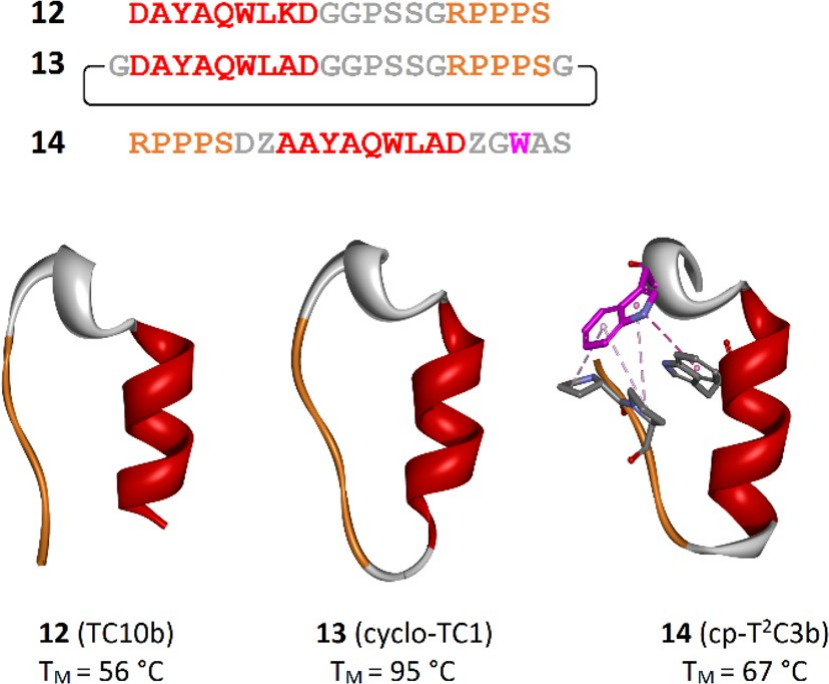
Sequences and NMR-derived structures of engineered Trp-cage folding constructs: acyclic (**12**), cyclized (**13**), and circularly permutated (**14**) forms. The Z letter stands for the Aib residue. The peptide backbone is presented as a ribbon. Red color denotes α-helix, orange is poly-Pro-helix, and gray are the loops and is consistent with coloring of the sequences. The side chains of the Trp residue introduced to restore the fold in 14 is shown as a stick in pink, and the hydrophobic interactions with this residue are depicted by dashed pink lines. All structures are present in the PDB database under the following codes: **12**, 2jof; **13**, 2ll5; **14**, 2m7c.

The interesting cases of protein engineering involve changing the connectivity of the secondary structures in the native sequence while retaining the overall fold. In one of the reports, the N- and C-termini of the stable Trp-cage construct (Figure 5, sequence **12**) were linked together by di-Gly peptide to produce cyclo-TC1 peptide (Figure 5, sequence **13**). This modification resulted in an increase of 35 °C in the melting point temperature compared to the acyclic form.^66^ Another study succeeded in the circular permutation of the Trp-cage fold.^67^ Circular permutation refers to cyclization of the tertiary structure and then breaking the continuity of the backbone at another position of the sequence. In this case, the cyclic and highly stable peptide mentioned above was used, and the new N- and C-termini were produced by cutting the original loop in between the α-helix and the poly-Pro-helix. As the original construct was poorly folded, several modifications previously reported for the ultrafast folding mutant^68^ were applied, and the Asp-Aib dipeptide instead of the Gly-Gly loop was introduced. This procedure restored the initial cyclic fold (Figure 5, sequence **14**), just with a lower *T*_m_ value (67 °C).

It is worth mentioning that the concept of protein engineering can be utilized not only for the construction of miniproteins but also for functionalizing the structures for chosen applications, especially in medicinal chemistry. Lying between small molecules and antibodies, they are easy to manufacture and administer and exhibit higher affinity due to the larger surface and can be promising in obtaining drugs for previously “undruggable” targets. The nice example is provided by the engineering of a miniature protein based on an avian pancreatic polypeptide and folding to a stable tertiary structure of a hairpin composed of a poly-Pro-helix and an α-helix. This short sequence was altered to bind proteins^69^ and nucleic acids.^70^ Examples include Bcl-2 and Bcl-XL binders obtained by grafting on the scaffold residues found on their native partner—the pro-apoptotic Bak protein.

Many miniproteins have been proven to form oligomers or even aggregates in solution, which may suppress their use as functional scaffolds. However, there are successful reports of sequence engineering that can promote monomerization, such as in the case of the avian pancreatic polypeptide.^71^ Native peptide forms a dimer at a micromolar concentration stabilized by π-stacking of aromatic residues presented on the surface. An alanine scan revealed that mutations of these residues lower the self-association tendency but also impact an overall fold. However, the restoration of the fold was possible by moving the Pro residue from position 13 to 14, which is observed in native sequences. This Pro residue is believed to be a poly-Pro-helix breaker and defines the relative orientation of poly-Pro- and α-helices.

## Incorporation of Noncanonical Amino Acids

The field of miniprotein design based on native folds was greatly expanded by the development of foldamer chemistry.^72,73^ Since the late 1990s, many secondary structures formed by peptidomimetic sequences were obtained, and the rules for designing helices, strands, and turns have been well-described.^74−76^ Recently, the concept of joining stable amphiphilic secondary structures together was applied for noncanonical secondary structures incorporating constrained β-amino acid. The helix–loop–helix structures were built from 9/12/9/10-helices containing *cis*-2-aminocyclopentanecarboxylic acid (*cis*-ACPC) amino acids and a flexible oligo-Gly linker.^77,78^ This rationale was also used to construct the foldameric helix–turn–helix structure.^79^ The use of a very rigid turn required a combinatorial calculation of possible spatial arrangements of the secondary structures based on experimental data of the torsions within the helix and the extended structure of the turn. The compactness of the structure was confirmed by 2D NMR.

**Figure 6:**
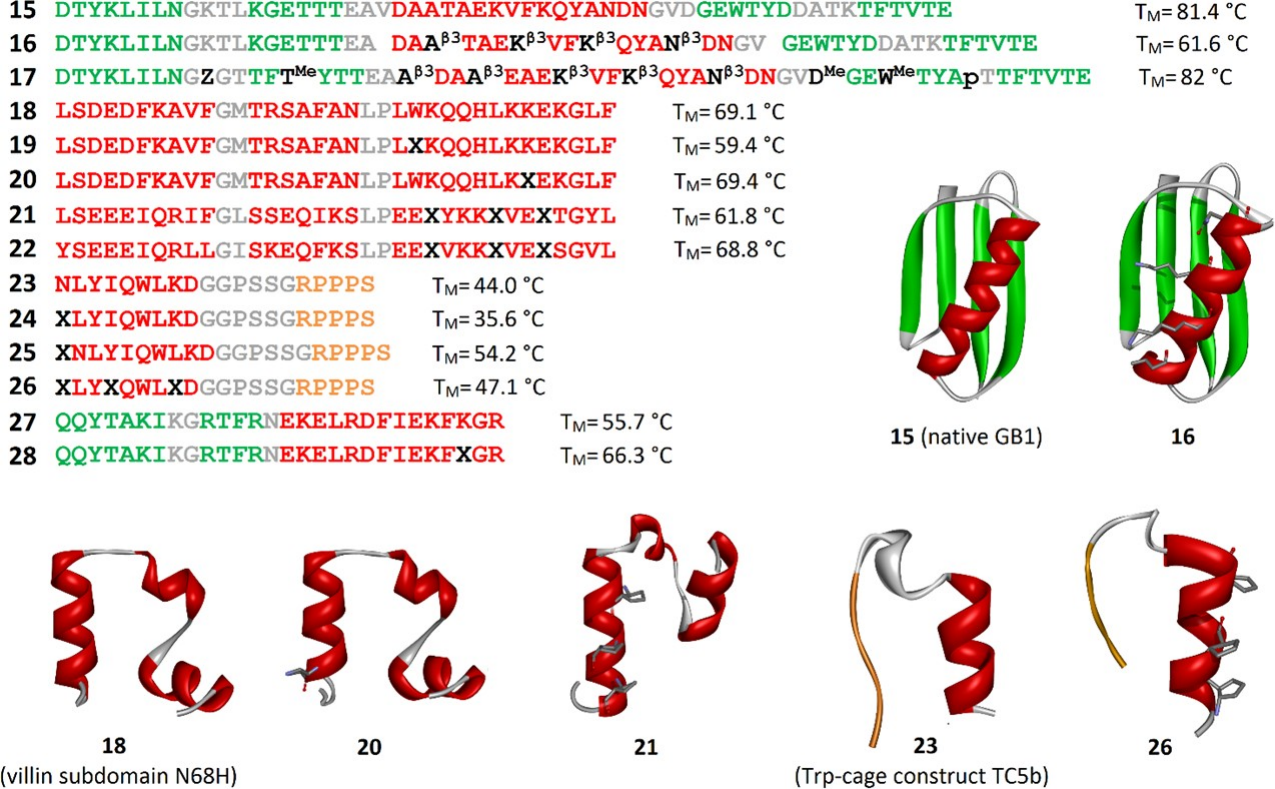
Sequences and, if available, experimentally derived structures of chosen noncanonical amino acids containing miniproteins. The peptide backbone is presented as a ribbon. The red color denotes α-helix, the orange color denotes the poly-Pro-helix, green is extended structure, and the gray color denotes loops and is consistent with the coloring of the sequences. The noncanonical residues are marked in black in the sequences and shown as gray sticks in the structures. The abbreviations of noncanonical residues are described in Figure 7. All structures, except **26** are present in the PDB database under the following codes: **15**, 3gb1; **16**, 4kgr; **18**, 1yrf; **20**, 5uil; **21**, 7ars; **23**, 1l2y.

Rational redesign of natural folds with noncanonical building blocks (referred to as heterogeneous backbone engineering^80^ or “foldamerization”^16^) led to the obtaining of a short and stable B1 domain of the G protein (GB1),^81^ N-terminal fragment of the villin headpiece (VHP),^82^ Trp-cage-like folds,^83^ or zinc finger folds.^84^ Extensive studies on the backbone modification were carried out in GB1 (Figure 6, sequence **15**) with the aim of changing approximately 20% of the sequence with heterogeneous building blocks but still maintaining the tertiary fold.^85^ In the iterative approach, the secondary structures were altered in the following manner: helix and loops forming residues were mutated into homologous β^3^-residues (carbon atom insertion); turns were substituted by C_α_-Me-α-residues or d-Pro and sheet-forming residues into N-Me-α-residues (Figure 6, sequences **16** and **17**). The peptide containing all of these mutations still adopts a tertiary structure and retains the conformational stability of the original fold.

The impact of the substitution of α-amino acid residues by β-amino acid analogues on the tertiary fold was also studied for the native VHP (Figure 6, sequence **18**). VHP is a 35-residue polypeptide that folds into the structure built from three short helical fragments that form the hydrophobic core. The first studies tested the single modification of the four solvent-exposed residues of the helical fragments.^86^ Replacements involved β^3^-amino acids with side chains homologous to the original α-amino acids or cyclopentane-based β-amino acids (*trans*-(3*R*,4*S*)-4-aminopyrrolidine-3-carboxylic acid (APC) for charged residues and *trans*-(1*S*,2*S*)*-*2-aminocyclopentanecarboxylic (*trans*-ACPC) for uncharged ones). Like in the case of GB1, structural studies showed that the overall tertiary structure of the modified peptides is well-maintained, but the majority of modifications lead to the destabilization of the fold, as indicated by a lower melting point. Only an analogue with mutation at the end of the helical fragment did not affect the conformational stability of the miniprotein (Figure 6, sequence **20**). In another study, the VHP sequence was modified not only by incorporation β-amino acids but also by computational redesign of α-residues.^82^ The *trans*-ACPC units were introduced into the longest C-terminal helix at positions consistent with previously described βαααβααβ motif.^87^ The α-residues were changed using the Rosetta FastDesign protocol^52^ to optimize the packing of the hydrophobic core (Figure 6, sequences **21** and **22**). Four out of five synthesized peptides were shown to fold cooperatively according to CD and showed *T*_m_ values close to the reference structure (VHP). However, the crystal structure obtained for one of them revealed, contrary to previous studies, a novel tertiary fold that was only slightly different from the original VHP (Figure 6, sequence **21**). Furthermore, peptide **21** was proven to form dimers both in the solution and in the solid state.

A slightly different approach, which eventually succeeded in obtaining altered sequences with conformational stability higher than the initial ones, was based on the systematic scan of the helical regions with the *trans*-ACPC residues. One of the modified sequences was the native Trp-cage motif, whereas the other was a zinc-finger-like FSD-1 sequence designed by Mayo and co-workers.^47^ The first round of the design involved single α → β mutations. Surprisingly, the observed sequence-stability relationships patterns were different in the case of Trp-cage-like sequences and FSD-1 mutants. Analogues of Trp-cage retain conformational stability similar to that of the initial sequence if the *trans*-ACPC residue did not interact directly with the hydrophobic core of the miniprotein. In case of FSD-1, only mutations near the C-terminus showed conformational stability close to the reference structure. However, in both cases, it was possible to identify mutants with a remarkably higher *T*_m_ value. In the next round, the whole helix was replaced by various implementations of βαααβααβ motif.^87^ Despite the numerous mutations, peptide **26** exhibited a *T*_m_ value close to that of the native Trp-cage. Detailed studies on the thermodynamics of folding have proven that constraining the helical structure with *trans*-ACPC residues has a beneficial entropic effect but causes an unfavorable enthalpy change. The NMR-derived structure of **26** shows a compact packing of the hydrophobic residues around the central Trp residue and a well-defined helical fragment with solvent-exposed *trans*-ACPC residues (Figure 6, sequence **26**).

**Figure 7:**
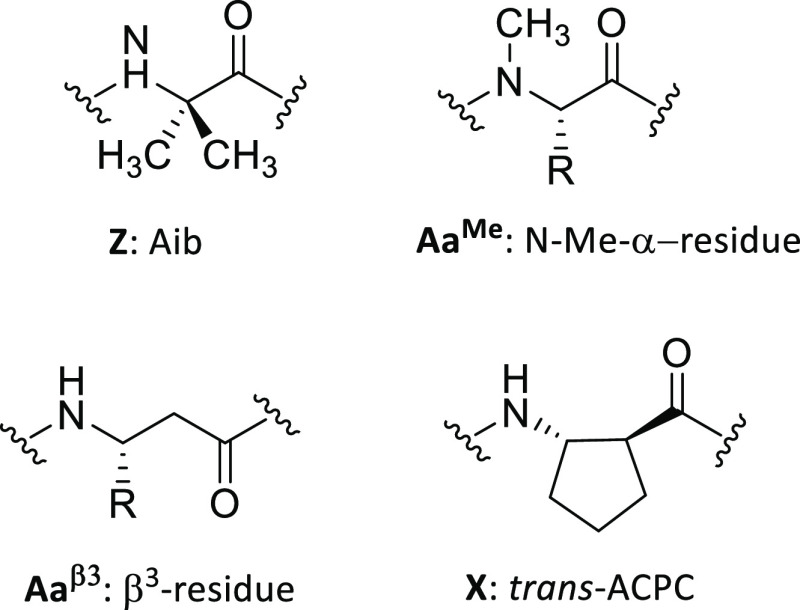
Noncanonical amino acid residues used for miniprotein engineering (Figure 5 and 6). The R group denotes the side chain of homologous α-amino acids.

## Conclusions and Perspectives

The field of miniproteins has changed significantly over the last 30 years and great progress has been achieved. Initial studies were based on very general rules and common sense, while recent successful contributions are mainly relying on efficient computer-aided methodologies. Numerous de novo designs of miniproteins of various geometries and secondary structure composition were described. Although high computing power and fast algorithms are available, often numerous molecules have to be obtained and evaluated to reach the expected output. Therefore, there is still considerable space for improvement of design algorithms.

Furthermore, miniproteins with modifications of side chains or backbone (e.g., incorporation of β-amino acid residues or N-methylation) have also been developed.^77,90,91^ The change of the main chain usually causes formation of non-native secondary structures, thus the number of possible miniprotein geometries in comparison to native counterparts increases considerably. Moreover, structures that are structurally distant from native miniproteins but also based on folding oligomeric molecules, have been also developed. These includes aromatic oligoamides or oligoureas that were shown to adopt miniprotein-like arrangements.^88,89^ These new directions of research will extend and inevitably deliver new structures in the future.

The world of miniprotein structures is constantly growing. The availability of structurally diversified miniproteins has also given the possibility of the development of functional molecules, in particular with biological activity. Although some examples have been published, the structural complexity and diversity of miniproteins provides great opportunities for the creation of structures with any desired function. Thus, over recent years the science of miniprotein has maturated and numerous research fields are open for further exploration.

## Abbreviations

CD: circular dichroism
SEC: size-exclusion chromatography
Aib: 2-aminoisobutyric acid
Pya: 3-(1,10-phenanthrol-2-yl)-l-alanine
*cis*-ACPC: *cis*-2-aminocyclopentanecarboxylic acid
trans-ACPC: *trans*-(1*S*,2*S*)*-*2-aminocyclopentanecarboxylic acid
APC: *trans*-(3*R*,4*S*)-4-aminopyrrolidine-3-carboxylic acid
GB1: B1 domain of protein G
VHP: villin headpiece subdomain
